# Association between physical activity and health literacy in patients with Parkinson’s disease: an online web survey

**DOI:** 10.1186/s12883-023-03437-7

**Published:** 2023-11-13

**Authors:** Koichi Nagaki, Takayasu Mishima, Tomoko Ohura, Kanako Kurihara, Shinsuke Fujioka, Yoshio Tsuboi

**Affiliations:** 1 Department of Neurology, Faculty of Medicine, Fukuoka University, 7-45-1 Nanakuma, Johnan-Ku, Fukuoka, 814-0180 Japan; 2 Center for Gerontology and Social Science, Research Institute, National Center for Geriatrics and Gerontology, 7-430, Morioka-cho, Obu City, Aichi 474-8511 Japan

**Keywords:** Parkinson’s disease, Health literacy, Physical activity

## Abstract

**Background:**

For patients with Parkinson's disease (PwPD), promotion of habitual physical activity (PA) assists in the prevention of disease progression. Patients' health literacy (HL) is integral for meeting PA standards and turning it into a habit. This study evaluated the association between PA level and each HL domain in PwPD.

**Methods:**

Online web-based assessment instruments and self-administered questionnaires, including the PA Questionnaire (IPAQ) Short Form and the Functional, Communicative, and Critical Health Literacy (FCCHL) scale, were used to assess PA levels and health literacy domains of PwPD.

**Results:**

The mean age of PwPD (*n* = 114) was 65.9 (SD = 11.6) years; 59.6% female, and the mean duration of disease was 6.4 (SD = 5.1) years. Of participants, 47.4% met the recommended criteria for PA. When comparing each HL domain by PA level, participants with lower PA had significantly lower critical HL (*p* = 0.03). Logistic regression analysis revealed that PA level correlated with critical HL (OR = 2.46; 95% CI = 1.16–5.19; *p* = 0.02).

**Conclusions:**

Adherence to recommended PA standards was associated with critical HL, but not other HL domains. Proactive attitudes to critically evaluate and utilize as well as understand health information may positively influence the promotion of PA.

## Background

Health literacy (HL) is a means of maintaining and promoting one's health and refers to an individual's ability to access, understand, evaluate, and apply health information [1]. Recently, this has been recognized as an important factor in maintaining health in adults and the elderly [2, 3]. There is also a rapidly growing body of evidence showing the impact of reduced HL on health outcomes in patients with chronic diseases such as Parkinson's disease (PD), which has been suggested to lead to reduced self-management [4–6]. The basic treatment strategy for PD is to promote appropriate medication and exercise/physical activity (PA). In both treatments, it is important for people with PD (PwPD) themselves to continuously self-manage their daily lives, in addition to following direct support from medical staff. Educational support is significant in promoting self-management for PwPD [7]. Given that PwPD face a wide variety of complex symptoms and treatment options, patient education is essential to ensure an understanding and acceptance of their disease; furthermore, differences in understanding and initiative should be considered when providing this education to specific patient populations. The benefits of combining patient education with medical therapy have been shown to reduce treatment costs, improve patient outcomes, and have positive effects on patient adherence to medication timing protocols [8, 9]. Patient education should therefore provide an ongoing teaching and learning process that involves a multidisciplinary team relevant to the patient in that particular setting [10].

Habitual promotion of exercise and PA may slow disease progression in the long term [11, 12]; knowing this, the World Health Organization (WHO) has published reference values for PA in chronic diseases such as PD, and it is hoped that this indicator will increasingly be recognized for PwPD [13]. However, exercise and PA require proactive behavior, which is often difficult to sustain. Indeed, it has been reported that PwPD often fail to adopt behaviors even when they recognize the need to engage in habitual PA [14]. Previous studies that have found an association between HL and PA have identified an association in patients with chronic diseases such as cardiovascular disease [15] and chronic kidney disease [16]. Educational interventions aimed at improving HL have also been shown significantly to impact patient health indicators and quality of life (QOL) in various chronic diseases, including type 2 diabetes [17] and ischemic heart disease [18]. However, existing literature on the impact of educational interventions for chronically ill patients shows that some subgroups respond to educational interventions and others do not [19]. In fact, there is no consensus on the association between PD medication knowledge/adherence and QOL [20, 21].

Previous studies have assessed the HL characteristics of PwPD, but this relationship between HL and PA remains controversial. Under one measure, PwPD HL was reported to be low, while under another measure several HL scores were reported to be at almost acceptable levels [22]. The focus of the index has been on "understanding." However, the concept of HL is more complex [23]; therefore, given the possibility that different domains may have other associations with HL, study results may need to be more consistent. To clarify the actual association of PwPD with PA, PD should be investigated with the addition of HL factors from other domains. The results of such a study could lead to additional cohort studies that further investigate causal relationships. In other words, each of these HL domains should be evaluated comprehensively and individually as they have different capabilities.

In PD, there are still few studies related to HL. To successfully implement patient self-management, a better understanding of the relationship between the HL component of PwPD and PA is needed. This will contribute to the promotion of PA based on self-management. Therefore, this study set out to determine the relationship between HL and PA levels. The relevance of each HL domain is very interesting and has yet to be investigated. It is hypothesized that the communicative and critical HL domains, which require a higher level of HL competence, will be associated with PA level.

## Methods

### Study design and participants

This cross-sectional observational study included PwPD who visited the Parkinson's Disease Medical Center at Fukuoka University Hospital. A web link to the survey questionnaire instrument was provided in a flyer distributed in conjunction with registration for membership on the web platform; this is an information distribution service for PwPD by the Department of Neurology, Fukuoka University. Web platform enrollment created a consecutive sample because flyers were distributed to PwPD who had been seen by the center. The distributed flyer included instructions to register for the web platform and to take the survey; it was left to each individual to click on the link where the details were explained. A diagnosis of PD was made according to the Movement Disorder Society's clinical diagnostic criteria for PD [24], and only patients who met the criteria for a definite or probable diagnosis of PD were invited to register. Upon registration, an online link generated by Survey Monkey was sent to PwPD. The survey was administered to those registered between February 1, 2022 and December 28, 2022.

The survey was completely anonymized, and the privacy of the participants was fully guaranteed by the authors. After being informed that their data would only be used for statistical and scientific purposes, participants agreed to complete a questionnaire regarding sociodemographic information and the following measures relevant to physical activity level and health literacy. In total, 146 Japanese PwPD aged 20 years or older responded to the questionnaire; people with suspected PD-like diseases (e.g., progressive supranuclear palsy and corticobasal degeneration) were excluded. In addition, to ensure the reliability of the data, we also excluded respondents whose responses were determined to be unanswered or fraudulently answered (such as inconsistent or intentionally wrong answers).

### Ethical approval

This study was conducted according to the Declaration of Helsinki, and the research protocol was approved by the Ethics Committee of Fukuoka University School of Medicine (U21-10–005). Informed consent was obtained from all participants.

### Measurements

#### Physical activity level

PA level was assessed using the PA Questionnaire (IPAQ) Short Form [25, 26]. The questions enabled the assessment of PA level by providing information on the minutes spent in activity during any given time of the week. The information captured in the questionnaire reported various types of PA (low, moderate, and vigorous), including walking activity and time spent sitting on an average day, including work days; moderate activity (i.e., carrying light loads, cycling at a steady pace, exercising in the yard); and vigorous activity (i.e., heavy lifting objects, intense aerobic exercise, bicycling or treadmill) [27]. The IPAQ-SF algorithm was used to convert continuous data into categorical data (i.e., low, moderate, and high PA) [28]. Outcomes were calculated as weekly metabolic equivalents of tasks in minutes (MET min/week). Regarding the assessment of PA levels, the World Health Organization recommends a minimum of 150 min per week of moderate PA and/or 75 min per week of vigorous PA for health promotion in adults with Parkinson's disease and other conditions [13]. Participants were subdivided into two categories according to the IPAQ information as follows: good levels of PA that met the recommendations above ("physically active") and insufficient PA that did not ("sedentary") [29].

#### Health literacy

HL was assessed using the Functional, Communicative, and Critical Health Literacy (FCCHL) scale developed by Ishikawa et al. [30]. HL consists of three components: functional, communicative, and critical [18]. Functional HL refers to basic reading and writing skills. Communicative HL refers to the ability to actively participate in daily life, extract information from various forms of communication, and understand its meaning. Finally, critical HL refers to higher cognitive and literacy skills that can be used to extract information from various forms of communication, understand its meaning, and apply new information to changing situations. The FCCHL is a self-administered questionnaire used to assess functional, communicative, and critical HL skills in people with chronic diseases; it consists of 14 questions, rated on a scale of 1 (never) to 4 (often). The average score for each HL is calculated from all questions, with higher average scores indicating higher abilities.

#### Sociodemographic variables

Participants were asked to provide the name of their diagnosis, their age, gender, duration of PD, and education level.

### Statistical analysis

Participant characteristics were compared between physically active (MPA ≥ 150 min/week and/or VPA ≥ 75 min/week) and sedentary (MPA < 150 min/week and/or VPA < 75 min/week) groups. Student's t-test and Pearson's chi-square test were used for continuous (e.g., age, duration of illness) and categorical (e.g., gender, education) variables, respectively.

A single logistic analysis was performed to examine the association between PA and HL, with PA level (physically active or sedentary) as the dependent variable and the three items of the HL sub-scale between the two groups as independent variables, respectively.

Two models were created, a crude one and one with adjustment for covariates (age, gender, duration of illness, and education) as it has been noted in previous studies that age, gender, and duration of illness may influence PA [31]. In addition, HL was selected as a covariate because it may well be influenced by educational background [32]. All statistical analyses were performed using R v.4.1.1 (R core team), and the significance level was set at 5%.

## Results

Finally, data from 141 PwPD were included in this study, and 114 respondents were analyzed, excluding those who met the exclusion above criteria. Of the 114 participants (age 65 ± 11.6 years, 59.6% female), 60 (52.6%) had inadequate levels of physically activity ("sedentary"), and 54 (47.4%) were fully physically active ("physically active"). The sociodemographic characteristics of the sedentary and physically active participants are shown in Table 1.

**Table 1:** Demographic and clinical characteristics of the study participants

Variables	Overall		Sedentary		Physically active		*p-*value
	**(*n* = 114)**		**(*n* = 60)**		**(*n* = 54)**		
Age, years	65.88	(11.6)	64.65	(11.5)	67.11	(11.8)	0.26
Females, *n*	68	(59.6)	40	(58.8)	28	(41.2)	0.11
PD duration, years	6.4	(5.1)	6.6	(6.0)	6.2	(4.0)	0.65
Academic background							
Junior high school, *n*	2	(1.8)	2	(3.3)	0	(0.0)	0.06
High school, *n*	39	(34.2)	24	(40.0)	15	(27.8)	
Professional school, *n*	13	(11.4)	4	(6.7)	9	(16.7)	
Junior college, *n*	16	(14.0)	10	(16.7)	6	(11.1)	
University, *n*	40	(35.1)	20	(33.3)	20	(37.0)	
Graduate school, *n*	4	(3.5)	0	(0.0)	4	(37.0)	

Values are shown as mean (SD) or n (%). *PD* Parkinson’s disease

Although there was no significant difference in education (*p* = 0.06), the sedentary group tended to have more middle school graduates, while the physically active group had more postgraduate degrees.

*The mean (SD) of each HL sub-item is shown below. Functional HL was overall 2.99 (0.63), physically active group 3.06 (0.60), and sedentary group 2.93 (0.66). Communicative HL was overall 3.16 (0.48), physically active group 3.19 (0.48), and sedentary group 3.11 (0.47). Critical HL was overall 2.88 (0.57), physically active group 3.00 (0.51), and sedentary group 2.77 (0.60).*

**Fig. 1:**
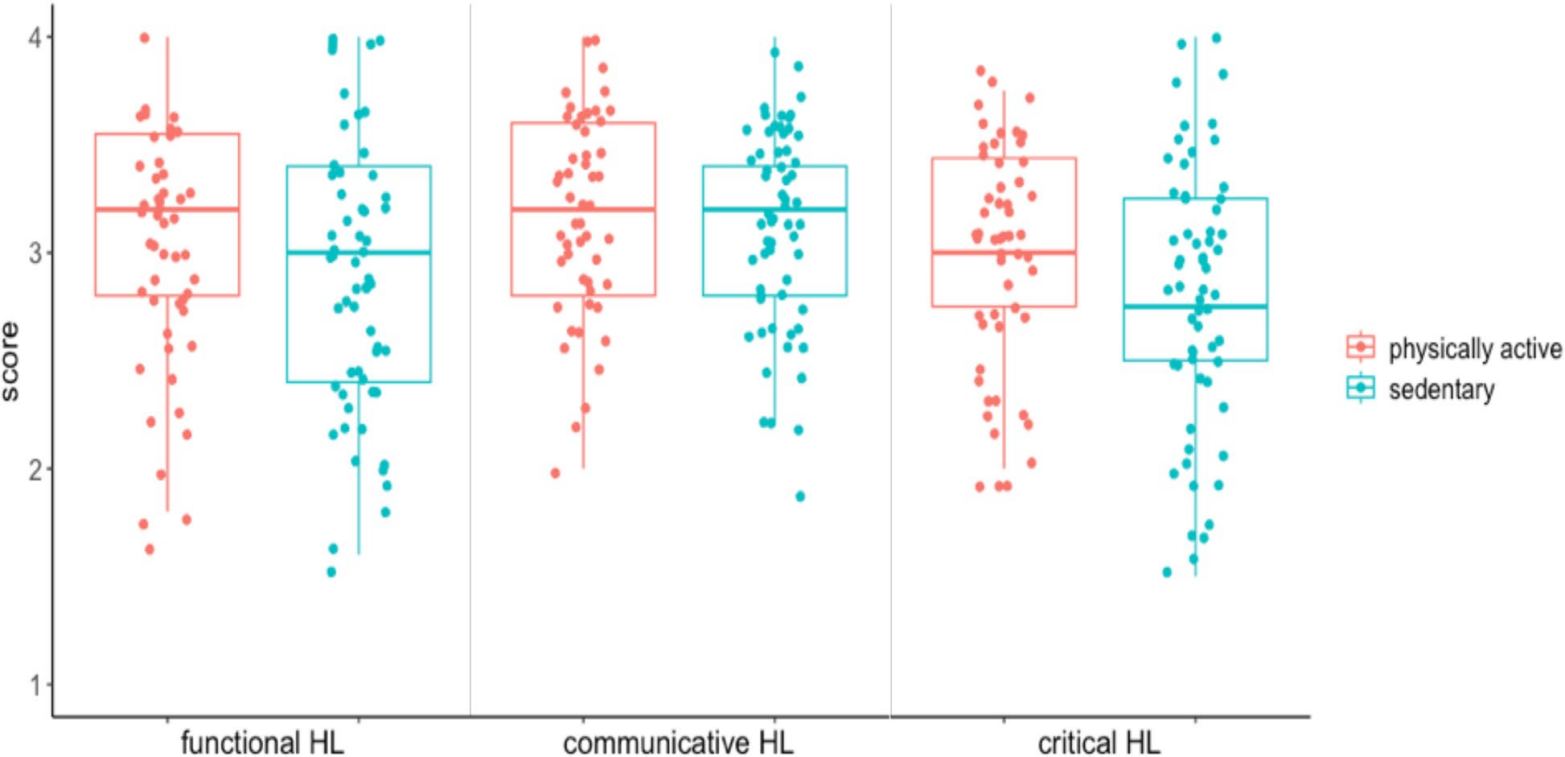
Comparison of health literacy by high and low physical activity

Figure 1 shows results comparing HL sub-items between the two groups.

There was no apparent difference between the two groups for functional HL (*p* = 0.21) and communicative HL (*p* = 0.44); for critical HL, the sedentary group had significantly lower results (*p* = 0.03) than the physically active group.

Table 2 shows the results of the single logistic regression analysis of PA and HL.

**Table 2:** Association between health literacy and physical activity by logistic regression analysis

Health Literacy sub-items	Crude Model			Adjusted Model		
	**OR**	**95% CI**	***p*-Value**	**OR**	**95% CI**	***p*-Value**
Functional	1.40	[0.77–2.53]	0.26	1.58	[0.84–2.96]	0.16
Communicative	1.42	[0.65–3.11]	0.38	1.51	[0.66–3.41]	0.33
Critical	2.12	[1.06–4.22]	0.03	2.46	[1.16–5.19]	0.02

Dependent variable: sedentary (MPA < 150 min/week and/or VPA < 75 min/week)

The covariate controlled for age, gender, PD duration, and the academic background

*OR* Odds ratio, *95% CI* 95% confidence interval of mean, *MVPA* Moderate to vigorous physical activity

The crude model (model not adjusted for covariates) showed that critical HL was associated with a decreasing regression (odds ratio (OR) = 2.12; 95% confidence interval (CI) = 1.06–4.22; *p* = 0.03). The adjusted model for covariates also demonstrated an association with lower critical HL (OR = 2.46; 95% CI = 1.16–5.19; *p* = 0.02), similar to the crude model.

## Discussion

Using an online survey, this study examined the association between PA and HL in PwPD. The results revealed that more than half of the participants did not meet the target PA criteria and that their physical inactivity was associated with critical HL. We believe that the ability to critically examine whether specific information about PA can be adapted to oneself, in addition to understanding and sharing the benefits of PA, can lead to the formation of exercise habits.

Few reports have evaluated the three aspects of HL separately and examined their association with PA in older adults and chronically diseased patients. Among them, the association between HL and PA in older adults suggests that although differences in HL does not change the number of steps taken, physical activity may be promoted by improving HL, thus allowing individuals to make beneficial health-related decisions on their own [33]. In addition, the knowledge, attitude, and practice (KAP) model proposes that when individuals receive relevant information, they develop an expected response, resulting in behaviors consistent with their attitudes [34]. However, outcomes and effectiveness do not necessarily coincide [35]. In other words, what one knows and what one does are not necessarily in line.

They also reported that one barrier to promoting PA in the long term is determining and presenting the optimal type and amount of exercise (frequency, intensity, etc.) [36]. However, there is still no clear consensus on the optimal amount of PA for people with chronic diseases, including PD, although the WHO has recently published guidelines [13].

Furthermore, PwPD have varying abilities depending on the presence and type of non-motor symptoms in addition to the individual's motor symptoms [37]. The fact that critical HL was associated with lower PA in the results of this study suggests that the ability to assess and utilize one's adaptation to the type and amount of PA was a factor in promoting PA.

In addition, this long-term enhancement of PA requires appropriate support from the health care provider to promote appropriate self-management; the promotion of self-management in PwPD includes receiving educational support, goal setting, and problem-solving suggestions from the health care provider [7]. Previous studies have shown that patient involvement in decisions about goals increases patient satisfaction [38], motivation [39, 40], and creates a sense of ownership [41]. Critical HL indicates a patient's confidence in critically examining information and adapting and utilizing it for his or her benefit. In other words, since proactive decision making is a key element, we infer that these elements of promoting self-management are based on critical HL.

From these results, it may be difficult for a system providing educational information focused on providing knowledge to promote PA in PwPD; however, health care providers have identified several communicative steps as a means of promoting HL [42]. These include using plain language, limiting and repeating information, using the teach-back method, and creating an atmosphere where people are not embarrassed to ask questions. This information also shows that if functional HL is not present, the ability of higher-order communicative and critical HL cannot be successfully utilized, so HL must be viewed both as its components and also comprehensively. In particular, the teach-back method provides a means of detecting patient misconceptions and confirming that they truly understand. A recent systematic review found that the teach-back method is also effective for health-related outcomes such as patient understanding, self-management, and quality of life [43]. In other words, it is hoped that the development of HL-related competencies on the part of professionals will, in turn, promote HL in patients.

There are several limitations to this study. First, it employed a cross-sectional study design and could not investigate changes in PA and HL, so we cannot refer to causal relationships. Second, since this study used an online survey, we must consider the possibility that the participants were PwPD with good access to the Internet and that most had education beyond high school (64.0%; See Table 1). In the present study, cases presenting with cognitive dysfunction were excluded, although no clear examination was available. Future studies should also include patients who present with cognitive dysfunction, which may require that caregivers also be included in the study. Third, HL varies between countries, with Japan showing low values [44], so its application to other countries should be interpreted cautiously. In addition, it is not easy to generalize results from online surveys. Finally, the inclusion of registrants of web platforms for educational information distribution in the survey, who are expected to be a more active audience for health information, may have introduced bias and should be further examined in more varied populations to reflect the target population adequately.

## Conclusions

We found an association between PA and critical HL in PwPD, and this association was not affected by age, gender, duration of illness, or educational history. These results suggest that patients' skills in assessing and utilizing their own health information may be important in promoting PA in PwPD. The present study was unable to demonstrate a causal relationship between PA and HL, but further confirmation is needed using a more representative multicenter-based sample or by cohort studies.

## Data Availability

The datasets used and/or analyzed during the current study available from the corresponding author on reasonable request.

## Abbreviations

FCCHL: Functional, Communicative, and Critical Health Literacy scale
HL: Health literacy
IPAQ: International Physical Activity Questionnaire
PA: Physical activity
PD: Parkinson’s disease
PwPD: Patients with Parkinson's disease
QOL: Quality of life
WHO: World Health Organization

## References

[1] Sørensen K, Van den Broucke S, Fullam J, Doyle G, Pelikan J, Slonska Z, Brand H (2012). (HLS-EU) Consortium Health Literacy Project European, Health literacy and public health: a systematic review and integration of definitions and models. BMC Public Health.

[2] Baker DW (2006). The meaning and the measure of health literacy. J Gen Intern Med.

[3] Nutbeam D (2008). The evolving concept of health literacy. Soc Sci Med.

[4] Berkman ND, Sheridan SL, Donahue KE, Halpern DJ, Crotty K (2011). Low health literacy and health outcomes: an updated systematic review. Ann Intern Med.

[5] Vandenbosch J, Van den Broucke S, Vancorenland S, Avalosse H, Verniest R, Callens M (2016). Health literacy and the use of healthcare services in Belgium. J Epidemiol Community Health.

[6] Bostock S, Steptoe A (2012). Association between low functional health literacy and mortality in older adults: longitudinal cohort study. BMJ.

[7] Kessler D, Liddy C (2017). Self-management support programs for persons with Parkinson’s disease: An integrative review. Patient Educ Couns.

[8] Montgomery EB, Lieberman A, Singh G, Fries JF (1994). Patient education and health promotion can be effective in Parkinson’s disease: A randomized controlled trial. Am J Med.

[9] Grosset KA, Grosset DG (2007). Effect of educational intervention on medication timing in Parkinson’s disease: a randomized controlled trial. BMC Neurol.

[10] van der Marck MA, Kalf JG, Sturkenboom IHWM, Nijkrake MJ, Munneke M, Bloem BR (2009). Multidisciplinary care for patients with Parkinson’s disease. Parkinsonism Relat Disord.

[11] Rafferty MR, Schmidt PN, Luo ST, Li K, Marras C, Davis TL, Guttman M, Cubillos F, Simuni T (2017). all NPF-QII Investigators, Regular Exercise, Quality of Life, and Mobility in Parkinson’s Disease: A Longitudinal Analysis of National Parkinson Foundation Quality Improvement Initiative Data. J Parkinsons Dis.

[12] Tsukita K, Sakamaki-Tsukita H, Takahashi R (2022). Long-term Effect of Regular Physical Activity and Exercise Habits in Patients With Early Parkinson Disease. Neurology.

[13] F.C. Bull, Al-Ansari S.S, Biddle S, Borodulin K, Buman M.P, Cardon G, Carty C, Chaput J.-P, Chastin S, Chou R, Dempsey P.C, DiPietro, L, Ekelund U, Firth J, Friedenreich  C.M, Garcia L, Gichu M, Jago R, Katzmarzyk P.T, Lambert E, Leitzmann M, Milton K, Ortega F.B, Ranasinghe C, Stamatakis E, Tiedemann A, Troiano R.P, van der Ploeg H.P, Wari V, Willumsen J.F (2020). World Health Organization 2020 guidelines on physical activity and sedentary behaviour. Br. J. Sports Med..

[14] Mantri S, Wood S, Duda JE, Morley JF (2019). Understanding physical activity in Veterans with Parkinson disease: A mixed-methods approach. Parkinsonism Relat Disord.

[15] Aaby A, Friis K, Christensen B, Rowlands G, Maindal HT (2017). Health literacy is associated with health behaviour and self-reported health: A large population-based study in individuals with cardiovascular disease. Eur J Prev Cardiol.

[16] Kita Y, Machida S, Shibagaki Y, Sakurada T (2021). Fact-finding survey on health literacy among Japanese predialysis chronic kidney disease patients: a multi-institutional cross-sectional study. Clin Exp Nephrol.

[17] Dwinger S, Rezvani F, Kriston L, Herbarth L, Härter M, Dirmaier J (2020). Effects of telephone-based health coaching on patient-reported outcomes and health behavior change: A randomized controlled trial. PLoS ONE.

[18] Eckman MH, Wise R, Leonard AC, Dixon E, Burrows C, Khan F, Warm E (2012). Impact of health literacy on outcomes and effectiveness of an educational intervention in patients with chronic diseases. Patient Educ Couns.

[19] Edlind M, Mitra N, Grande D, Barg FK, Carter T, Turr L, Glanz K, Long JA, Kangovi S (2018). Why Effective Interventions Do Not Work for All Patients: Exploring Variation in Response to a Chronic Disease Management Intervention. Med Care.

[20] H.M. Zipprich, S. Mendorf, T. Lehmann, T. Prell, Self-Reported Nonadherence to Medication Is Not Associated with Health-Related Quality of Life in Parkinson’s Disease, Brain Sci. 11 (2021). 10.3390/brainsci11020273. 10.3390/brainsci11020273 PMC7926683 33671679

[21] Zipprich HM, Mendorf S, Schönenberg A, Prell T (2022). The impact of poor medication knowledge on health-related quality of life in people with Parkinson’s disease: a mediation analysis. Qual Life Res.

[22] Fleisher JE, Shah K, Fitts W, Dahodwala NA (2016). Associations and implications of low health literacy in Parkinson’s Disease. Mov Disord Clin Pract.

[23] Nutbeam D (2000). Health literacy as a public health goal: a challenge for contemporary health education and communication strategies into the 21st century. Health Promot Int.

[24] Postuma RB, Berg D, Stern M, Poewe W, Olanow CW, Oertel W, Obeso J, Marek K, Litvan I, Lang AE, Halliday G, Goetz CG, Gasser T, Dubois B, Chan P, Bloem BR, Adler CH, Deuschl G (2015). MDS clinical diagnostic criteria for Parkinson’s disease. Mov Disord.

[25] Craig CL, Marshall AL, Sjöström M, Bauman AE, Booth ML, Ainsworth BE, Pratt M, Ekelund U, Yngve A, Sallis JF, Oja P (2003). International physical activity questionnaire: 12-country reliability and validity. Med Sci Sports Exerc.

[26] Lee PH, Macfarlane DJ, Lam TH, Stewart SM (2011). Validity of the International Physical Activity Questionnaire Short Form (IPAQ-SF): a systematic review. Int J Behav Nutr Phys Act.

[27] Maugeri G, Castrogiovanni P, Battaglia G, Pippi R, D’Agata V, Palma A, Di Rosa M, Musumeci G (2020). The impact of physical activity on psychological health during Covid-19 pandemic in Italy. Heliyon.

[28] C. Forde, Scoring the international physical activity questionnaire (IPAQ), (2018). https://ugc.futurelearn.com/uploads/files/bc/c5/bcc53b14-ec1e-4d90-88e3-1568682f32ae/IPAQ_PDF.pdf. Accessed 24 Jan 2023.

[29] Piercy KL, Troiano RP, Ballard RM, Carlson SA, Fulton JE, Galuska DA, George SM, Olson RD (2018). The Physical Activity Guidelines for Americans. JAMA.

[30] Ishikawa H, Takeuchi T, Yano E (2008). Measuring functional, communicative, and critical health literacy among diabetic patients. Diabetes Care.

[31] van Nimwegen M, Speelman A.D, Hofman-van Rossum E.J.M, Overeem S, Deeg D.J.H, Borm G.F, van der Horst M.H.L, Bloem B.R, Munneke M (2011). Physical inactivity in Parkinson’s disease. J. Neurol.

[32] Paasche-Orlow MK, Parker RM, Gazmararian JA, Nielsen-Bohlman LT, Rudd RR (2005). The prevalence of limited health literacy. J Gen Intern Med.

[33] Lim ML, van Schooten KS, Radford KA, Delbaere K (2021). Association between health literacy and physical activity in older people: a systematic review and meta-analysis. Health Promot Int.

[34] Allport GW. A handbook of social psychology. Clark University Press; 1935. p. 798–844.

[35] Bandura A (2001). Social cognitive theory: an agentic perspective. Annu Rev Psychol.

[36] Schootemeijer S, van der Kolk NM, Ellis T, Mirelman A, Nieuwboer A, Nieuwhof F, Schwarzschild MA, de Vries NM, Bloem BR (2020). Barriers and Motivators to Engage in Exercise for Persons with Parkinson’s Disease. J Parkinsons Dis.

[37] K. Nagaki, S. Fujioka, H. Sasai, Y. Yamaguchi, Y. Tsuboi, Physical Activity and Its Diurnal Fluctuations Vary by Non-Motor Symptoms in Patients with Parkinson’s Disease: An Exploratory Study, Healthcare (Basel). 10 (2022). 10.3390/healthcare10040749. 10.3390/healthcare10040749 PMC9029803 35455926

[38] L. Turner-Stokes, H. Rose, S. Ashford, B. Singer, Patient engagement and satisfaction with goal planning: Impact on outcome from rehabilitation, Int. J. Ther. Rehabil. 22 (2015). https://www.researchgate.net/profile/Barbara-Singer-2/publication/276267359_Patient_engagement_and_satisfaction_with_goal_planning_Impact_on_outcome_from_rehabilitation/links/555ab40008ae6fd2d8283579/Patient-engagement-and-satisfaction-with-goal-planning-Impact-on-outcome-from-rehabilitation.pdf.

[39] Van De Weyer RC, Ballinger C, Playford ED (2010). Goal setting in neurological rehabilitation: staff perspectives. Disabil Rehabil.

[40] Leach E, Cornwell P, Fleming J, Haines T (2010). Patient centered goal-setting in a subacute rehabilitation setting. Disabil Rehabil.

[41] Holliday RC, Ballinger C, Playford ED (2007). Goal setting in neurological rehabilitation: patients’ perspectives. Disabil Rehabil.

[42] Weiss BD. Help patients understand. Manual for Clinicians. American Medical Association Foundation. 2007. http://healthlinks.web-32.com/user/healthlinks/webimg/201249/Weiss%20-%20AMA%20Guide%20to%20Health%20Literacy.pdf.

[43] Talevski J, Wong Shee A, Rasmussen B, Kemp G, Beauchamp A (2020). Teach-back: A systematic review of implementation and impacts. PLoS One.

[44] Nakayama K, Osaka W, Togari T, Ishikawa H, Yonekura Y, Sekido A, Matsumoto M (2015). Comprehensive health literacy in Japan is lower than in Europe: a validated Japanese-language assessment of health literacy. BMC Public Health.

